# Supplementary material for: Temperature-Dependent Rheological and Viscoelastic Investigation of a Poly(2-methyl-2-oxazoline)-b-poly(2-iso-butyl-2-oxazoline)-b-poly(2-methyl-2-oxazoline)-Based Thermogelling Hydrogel
Source: J Funct Biomater. 2019 Aug 7;10(3):36. doi: 10.3390/jfb10030036 (PMC6787588; doi:10.3390/jfb10030036)
Supplement: Supplementary file 1 [file jfb-10-00036-s001.pdf]

Temperature dependent rheological and viscoelastic investigation of a poly(2-methyl-2-oxazoline)-b-poly(2-*iso*-butyl-2-oxazoline)-b-poly(2-methyl-2-oxazoline) based thermogelling hydrogel

Michael M. Lübtow[1,*], Miroslav Mrlik[1,2,*], Matthias Beudert[3], Tessa Lühmann[3], Robert Luxenhofer[1]

[1] Polymer Functional Materials, Chair for Advanced Materials Synthesis, Department of Chemistry and Pharmacy and Bavarian Polymer Institute, Julius-Maximilians-University Würzburg, Würzburg, Germany;

[2] Centre of Polymer Systems, University Institute, Tomas Bata University in Zlin, Trida T. Bati 5678, 760 01, Zlin, Czech Republic.

[3] Institute of Pharmacy and Food Chemistry, Julius-Maximilians-University Würzburg, Am Hubland, 97074 Würzburg, Germany

[*] both authors corresponded equally

Keywords: amphiphilic block copolymer, poly(oxazoline), viscoelasticity, Vocaldo-like behaviour, thermo-responsive hydrogel,

Correspondence to: robert.luxenhofer@uni-wuerzburg.de

Synthesis of 2-*iso*-butyl-2-oxazoline (*i*BuOx)

The synthesis of 2-*iso*-butyl-2-oxazoline (*i*BuOx), as the core forming, hydrophobic block was performed according to Witte and Seeliger[1] (Figure S1S) and described previously [2].

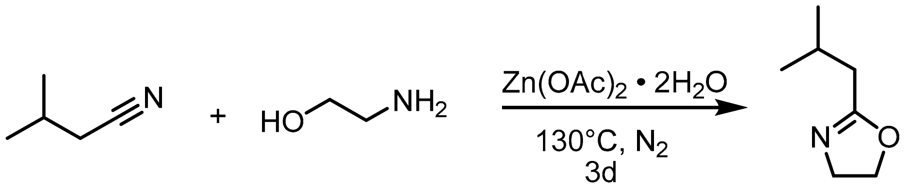

7.95 g of isovaleronitrile (95.6 mmol, 1 eq), 7.59 g of ethanolamine (124 mmol, 1.3 eq) and 0.52 g of zinc acetate dihydrate (2.39 mmol, 0.025 eq) were added to a nitrogen flushed flask and heated to 130 °C. The reaction continued under reflux for 3d until the reaction mixture turned brownish. Reaction progress was controlled by FTIR- and ^{1}H-NMR-spectroscopy. The raw product was dissolved in dichloromethane and washed with H_2O (3x). The organic phase was dried with $MgSO_4$, filtered and concentrated under vacuum. The residue was mixed with CaH_2 and distilled via vacuum distillation.

Yield	7.59 g (59.3 mmol; 62%) of a colorless liquid
bp	43 °C; 5 mbar
Mw	127.19 g/mol

^{1}H-NMR:	($CDCl_3$; 300.12 MHz; 298 K): δ = 4.16 (t, 2H, H^1, ^3J = 9.4 Hz); 3.78 (t, 2H, H^2, ^3J = 9.4 Hz); 2.10 (d, 2H, H^3); 2.00 (m, 1H, H^4); 0.93 (m, 3H, H^5)
^{13}C-NMR:	($CDCl_3$; 300.12 MHz; 298 K): δ = 167.9 (C^1); 67.1 (C^2); 54.4 (C^3); 37.0 (C^4); 26.3 (C^5); 22.5 (C^6)

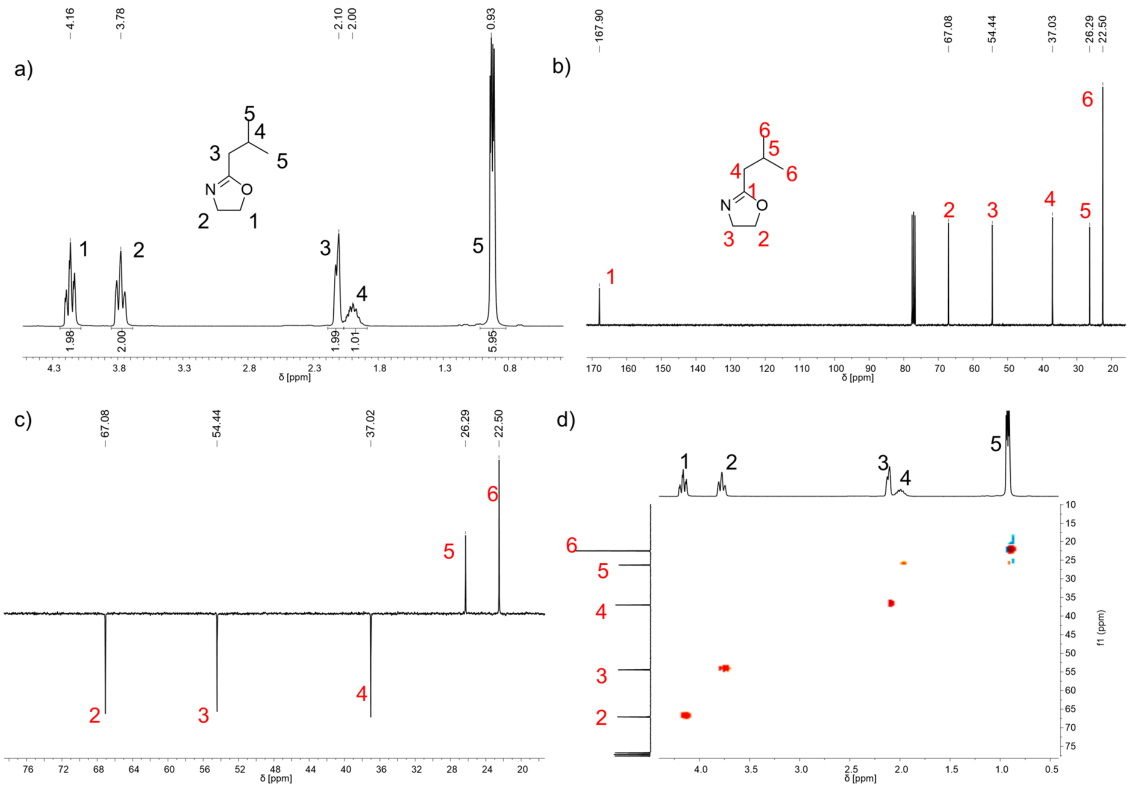

Figure S1. a) ^{1}H-NMR; b) ^{13}C-NMR; c) DEPT-135 and d) HSQC ($CDCl_3$; 300 MHz; 298 K) of *i*BuOx with signal assignment of all major signals.

Me-MeOx$_{36}$-*i*BuOx$_{18}$-MeOx$_{32}$-PipBoc (A-p*i*BuOx-A)

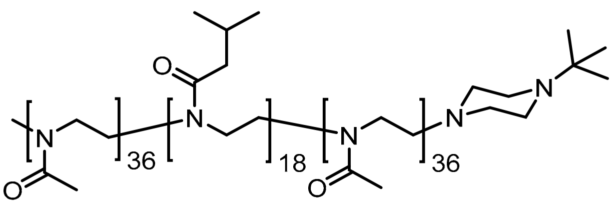

The polymerizations and work-up procedures were carried out as described previously.[3]
352 mg of methyl trifluoromethanesulfonate (2.14 mmol; 1 eq) and 6.56 g 2-methyl-2-oxazoline (MeOx) (77.1 mmol; 36 eq) were added to a dried and nitrogen flushed flask and dissolved in 43 mL benzonitrile (PhCN). The reaction mixture was heated to 100 °C for approximately 4 hours. Reaction progress was controlled by FTIR- and ^{1}H-NMR-spectroscopy. After complete consumption of MeOx, the mixture was cooled to RT and 5.0 g *i*BuOx (39.3 mmol; 18 eq) was added. The reaction mixture was heated to 100 °C overnight. 6.50 g MeOx (76.3 mmol; 36 eq) were added and the reaction stirred for 4h at 100 °C. Termination was carried out by addition of 1.22 g 1-Boc-piperazine (PipBoc) (6.6 mmol; 3 eq) at 50 °C for 4 hours. Subsequently, 296 mg of K_2CO_3 (2.13 mmol; 1 eq) was added and the mixture was stirred at 50 °C for 4 hours. Precipitates were removed by centrifugation and PhCN was removed under reduced pressure. The crude product was transferred to a dialysis bag (MWCO 1 kDa, cellulose acetate) and dialyzed against Millipore water overnight. The solution was recovered from the bag and lyophilized.

Yield	16.6 g (1.94 mmol; 90%) of a white powder
M_w	8.5 kg/mol
GPC (HFIP)	M_n = 4.3 kg/mol; Đ = 1.12
^{1}H-NMR	M_n = 8.2 kg/mol (Me-MeOx$_{36}$-*b*-*i*BuOx$_{18}$-*b*-MeOx$_{32}$-PipBoc)
	($CDCl_3$, 300.12 MHz; 298 K): δ = 3.81–3.10 (br, 345H, H^1); 3.07– 2.92 (br, 3H, H^2); 2.59–2.38 (br, 5H, H^3); 2.29–2.00 (br, 252H, $H^{4,5,6}$); 1.50–1.40 (s, 8H, H^7); 1.00–0.84 (br, 106H, H^8).

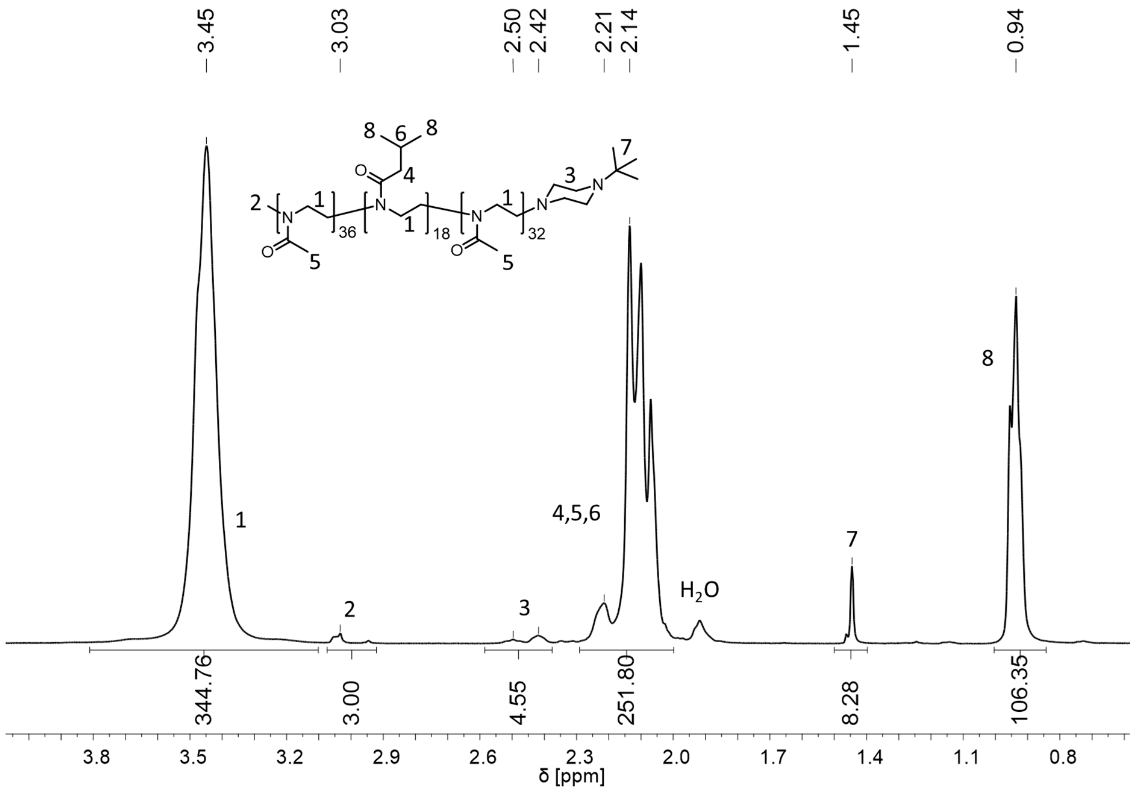

Figure S2. ^{1}H-NMR ($CDCl_3$; 300 MHz; 298 K) of **A-p*i*BuOx-A** with signal assignment of all major signals.

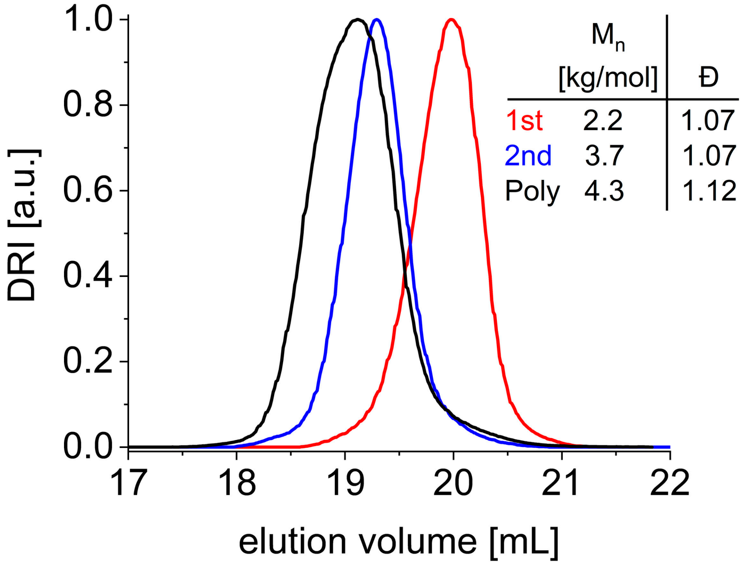

Figure S3. GPC elugrams (HFIP) of **A-p*i*BuOx-A** after each polymerized block and polymer after work-up.

References

[1] H. Witte, W. Seeliger, *Liebigs Ann. Chem.* **1974**, *1974*, 996.
[2] M. M. Lübtow, M. S. Haider, M. Kirsch, S. Klisch, R. Luxenhofer, *Biomacromolecules* **2019**, *in print*, DOI: 10.1021/acs.biomac.9b00618.
[3] R. Luxenhofer, R. Jordan, *Macromolecules* **2006**, *39*, 3509.
